# Absent lunula: An overlooked finding in chronic kidney disease

**DOI:** 10.1002/ccr3.3471

**Published:** 2020-11-20

**Authors:** Najla Daadaa, Asmahane Souissi, Badreddine Ben Kaab, Mohamed Karim Zouaghi, Mourad Mokni

**Affiliations:** ^1^ Dermatology Department La Rabta Hospital Tunis Tunisia; ^2^ Nephrology Department La Rabta Hospital Tunis Tunisia

**Keywords:** absent lunula, chronic kidney disease, hemodialysis

## Abstract

We highlight the importance of a thorough nail examination in every clinical encounter, especially in uremic patients. Absent lunula should prompt the clinician to rule out underlying kidney disease even in the absence of signs of uremia.

## Graphical abstract

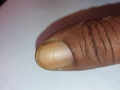

## 1 CASE HISTORY

Thorough nails examination should be an integral part of exhaustive physical examination in uremic patients since nail disorders, as absent lunula, can serve as a marker of chronic kidney disease in the absence of more alarming signs.

**FIGURE 1:**
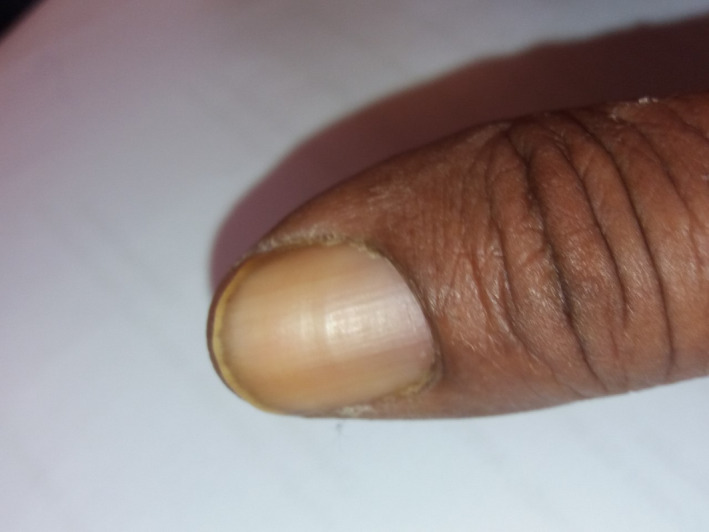
Absent lunula of the thumbnail

**FIGURE 2:**
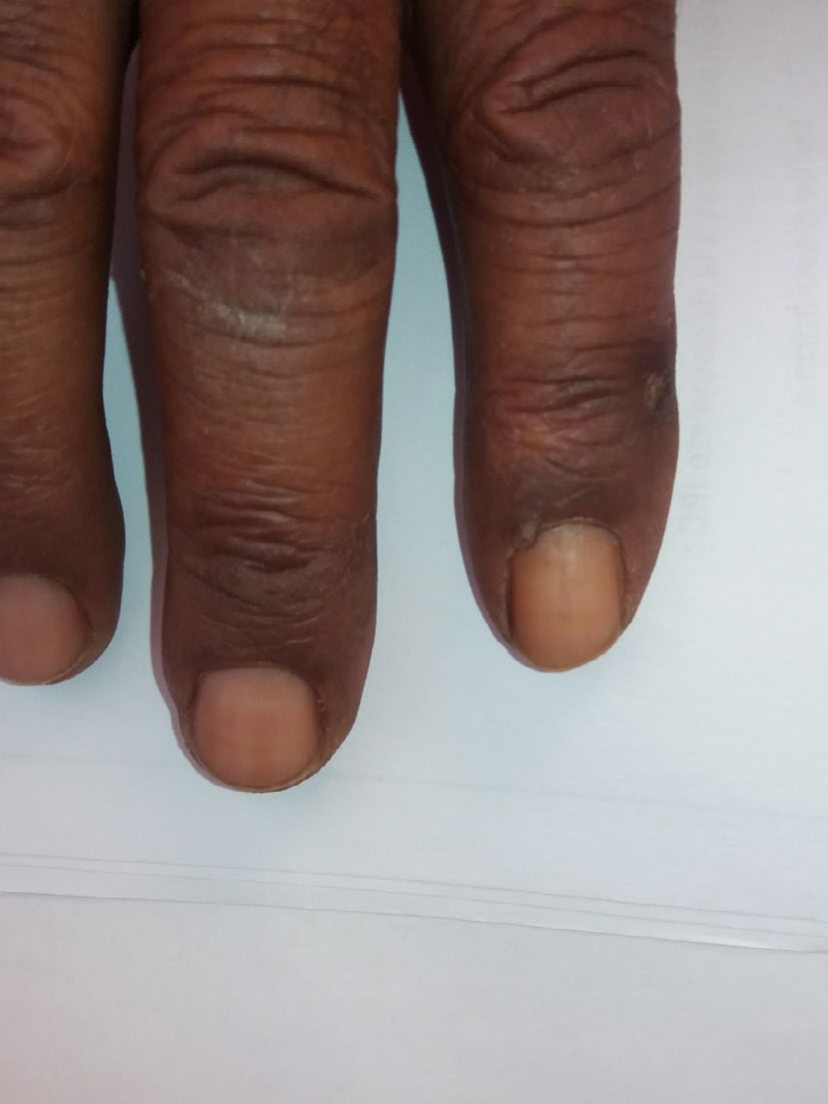
Absent lunula of the other fingernails

A 62‐year‐old man was addressed from the nephrology department for a systematic dermatological check‐up. He reported a history of an end‐stage chronic kidney disease (CKD) due to hypertensive kidney disease. He had not been on hemodialysis yet. On close observation, the lunula of all fingernails was surprisingly absent (Figures 1, 2and1, 2). Blood investigations revealed anemia (11 g/dL), hypocalcemia (73 mg/L), and low serum iron level (0.28 mg/L).

The lunula is the visible portion of the nail matrix that extends beyond the proximal nail fold.^1^ Absent lunula (AL) has been recorded in up to 62.9% of patients with CKD^2^ but also described in chronic obstructive pulmonary disease and rheumatoid arthritis.^1^ Anemia, seen in our patient, is thought to be a causative effect of AL.^3^ However, other reports suggested that it reflected rather a combination of several conditions in uremic patients.^2^, ^3^ AL has been observed before hemodialysis. Thus, it is believed that CKD itself, not particularly hemodialysis, could play a role in the development of AL.^2^

In conclusion, AL can serve as a marker of CKD in the absence of more alarming signs. Therefore, thorough nails examination should be an integral part of exhaustive physical examination in uremic patients.

## CONFLICT OF INTEREST

No conflict of interest.

## AUTHOR CONTRIBUTIONS

DN: helped in writing the manuscript, did literature search, and is corresponding author. AS: collected clinical data, conceptualized the article, and did final proofreading of the submission. KB: helped in writing manuscript and took clinical pictures. MKZ and MM: revised and approved the final version of the manuscript.

## Data Availability

Data sharing not applicable to this article as no datasets were generated or analyzed during the current study.
